# Graphene Oxide Nanosheets for Bone Tissue Regeneration

**DOI:** 10.3390/molecules29143263

**Published:** 2024-07-10

**Authors:** Jorge Iván Castro, Alana Payan-Valero, Carlos-Humberto Valencia-Llano, Mayra Eliana Valencia Zapata, Jose Herminsul Mina Hernández, Paula A. Zapata, Carlos David Grande-Tovar

**Affiliations:** 1 Tribology, Polymers, Powder Metallurgy and Solid Waste Transformations Research Group, Universidad del Valle, Calle 13 No. 100-00, Cali 760001, Colombia; jorge.castro@correounivalle.edu.co; 2 Grupo Biomateriales Dentales, Escuela de Odontología, Universidad del Valle, Calle 4B Número 36-00, Cali 760001, Colombia; alana.payan@correounivalle.edu.co (A.P.-V.); carlos.humberto.valencia@correounivalle.edu.co (C.-H.V.-L.); 3 Grupo de Materiales Compuestos, Escuela de Ingeniería de Materiales, Facultad de Ingeniería, Universidad del Valle, Calle 13 Número 100-00, Cali 760032, Colombia; valencia.mayra@correounivalle.edu.co (M.E.V.Z.); jose.mina@correounivalle.edu.co (J.H.M.H.); 4 Grupo de Polímeros, Facultad de Química y Biología, Universidad de Santiago de Chile, Santiago de Chile 9170020, Chile; paula.zapata@usach.cl; 5 Grupo de Investigación de Fotoquímica y Fotobiología, Universidad del Atlántico, Carrera 30 Número 8-49, Puerto Colombia 081008, Colombia

**Keywords:** bone tissue engineering, biocompatibility, degree oxidation, graphene oxide, bone implantation

## Abstract

Bone tissue engineering is a promising alternative to repair wounds caused by cellular or physical accidents that humans face daily. In this sense, the search for new graphene oxide (GO) nanofillers related to their degree of oxidation is born as an alternative bioactive component in forming new scaffolds. In the present study, three different GOs were synthesized with varying degrees of oxidation and studied chemically and tissue-wise. The oxidation degree was determined through infrared (FTIR), X-ray diffraction (XRD), X-ray photoelectron (XPS), and Raman spectroscopy (RS). The morphology of the samples was analyzed using scanning electron microscopy (SEM). The oxygen content was deeply described using the deconvolution of RS and XPS techniques. The latter represents the oxidation degree for each of the samples and the formation of new bonds promoted by the graphitization of the material. In the RS, two characteristic bands were observed according to the degree of oxidation and the degree of graphitization of the material represented in bands D and G with different relative intensities, suggesting that the samples have different crystallite sizes. This size was described using the Tuinstra–Koenig model, ranging between 18.7 and 25.1 nm. Finally, the bone neoformation observed in the cranial defects of critical size indicates that the F1 and F2 samples, besides being compatible and resorbable, acted as a bridge for bone healing through regeneration. This promoted healing by restoring bone and tissue structure without triggering a strong immune response.

## 1. Introduction

Modeling and reconstructing bone tissue is a response to exposed damage when fractures, skeletal development, or normal physiological remodeling occur [1]. Nevertheless, in recent years, tissue engineering has gained a leading role because it allows rapid revascularization, the induction of osteogenesis, and osteoinduction [2], causing the generation of new tissue with characteristics similar to those of the original tissue. It has advantages compared to traditional methods because they present lower healing speeds, donor morbidity, and immune complications, which must be considered [3]. Several biomaterials, such as natural or synthetic polymers, have been used in combination or individually to study their biocompatibility, biodegradability, proliferation, and cellular differentiation [4,5]. However, combining several polymers may or may not act synergistically for wound healing. Still, when the damage is severe, other properties such as chemical stability or thermal properties are necessary to stabilize the system and not degrade rapidly. Thus, nanomaterials and nanocomposites have been created as an alternative to overcome this problem.

As a consequence of the above, it has become necessary to develop different types of scaffolds that involve other systems different from those currently used, such as calcium phosphate (hydroxyapatite and ceramics), biodegradable polymers, and scaffolds based on carbon nanomaterials [6]. The latter has shown excellent mechanical properties, biocompatibility, good thermal conductivity, and high absorption capacity, making it an excellent candidate for the formation of scaffolds. Graphene is a 2D structure with sp^2^ hybridization similar to a honeycomb and is formed by a monolayer of carbon atoms [7]. Exfoliation and oxidation lead to epoxy groups, alcohols, and carboxylic acids forming within its structural plane, essential in various fields such as biosensors, designing optical devices operating in this frequency range [8], photoelectric devices [9], bioimaging, cancer therapy, tissue engineering, and drug delivery [10]. Their unique properties, such as high surface area, excellent mechanical strength, good elasticity pattern, antibacterial activity, good biocompatibility, and ease of functionalization [11,12], make them suitable for biological applications.

Despite graphene oxide (GO) offering promising applications across various biomedical domains, a comprehensive assessment of its toxicological impacts on health and the environment still needs to be discovered [13]. Human exposure to these carbon-based materials is inevitable daily, underscoring the urgency for extensive nanotoxicological investigations to gauge safety and potential risks. Past research has illuminated the intricate interactions between carbonaceous nanomaterials and various biological systems. Nevertheless, gaps persist in understanding their biocompatibility and interactions with the extracellular matrix in bone, presenting avenues for exploring novel alternatives.

GO has been used in polyhydroxybutyrate–chitosan polymeric matrices, demonstrating excellent mechanical properties due to the electrostatic π-π interaction and bonding between the oxygenated groups of the nanofiller and the polymeric matrix. Additionally, the addition of GO to the matrix increased the growth of MG-63 osteosarcoma cells due to its high surface area, presence of surface active groups, hydrophilic nature, and increased surface roughness at the nanoscale [14]. On the other hand, a 3D biomimetic framework was designed with cortical and spongy regions along with Havers channels, where it was demonstrated that the presence of GO nanoparticles changes the surface properties from hydrophobic to hydrophilic, increasing the wettability of the polycaprolactone, and GO composite scaffolds as well as the robust survival and strong adherence of MG-63 osteoblast cells demonstrate that PCL/GO composite scaffolds exhibit impressive biocompatibility, positioning them as an up-and-coming option for bone tissue engineering endeavors [15]. Finally, GO-modified sodium alginate/polyethylene glycol phase-change materials have been formed for temperature control during photothermal therapy, where upon adding 0.5% GO, the material boasts a substantial thermal energy storage capacity of 100. 0 J/g and a commendable latent heat recovery rate of 93.3%, ideal for thermal management and temperature control, but it also exhibits a suitable phase-change temperature of 42.2 °C, facilitating bone formation [16].

Despite the numerous publications on the application of GO in tissue bone engineering, specific limitations exist that hinder its direct use. These include concerns regarding its biocompatibility and hemocompatibility, colloidal stability, and assessing its influence on the durability of bonding and the fusion of bone, among others [17]. Additionally, graphene-based nanomaterials hold significant value in tissue engineering and regenerative medicine. Their profound impact is particularly evident in facilitating stem cell proliferation, differentiation, and migration, thereby driving substantial advancements in tissue regeneration. The motivation for this research arises from the exceptional characteristics of GO, which exhibits remarkable specificity, exceptional chemical stability, compatibility with biological systems, and the capacity for functionalization, positioning it as an excellent candidate for diverse applications. However, it is essential to consider factors such as the material’s toxicity, concentration, surface chemistry, morphology, thickness, and purity, as these elements are intricately connected to GO synthesis [18]. To the best of our knowledge, no studies have evaluated the in vivo biocompatibility in bone through the implantation of different GOs in Wistar rats according to the oxidation degree of different GOs synthesized. The physicochemical characterization demonstrated the presence of carbonyl, hydroxyl, and epoxy groups in various proportions, confirming the samples’ oxidation. The biocompatibility exhibited by the GOs prepared here, besides the bone neoformation and increased nanosheet resorption, indicates a promising result for GO’s use for bone tissue regeneration, according to our results.

## 2. Results and Discussion

### 2.1. Characterization of Graphene Oxide

#### Fourier Transform Infrared Spectroscopy (FTIR)

**Figure 1:**
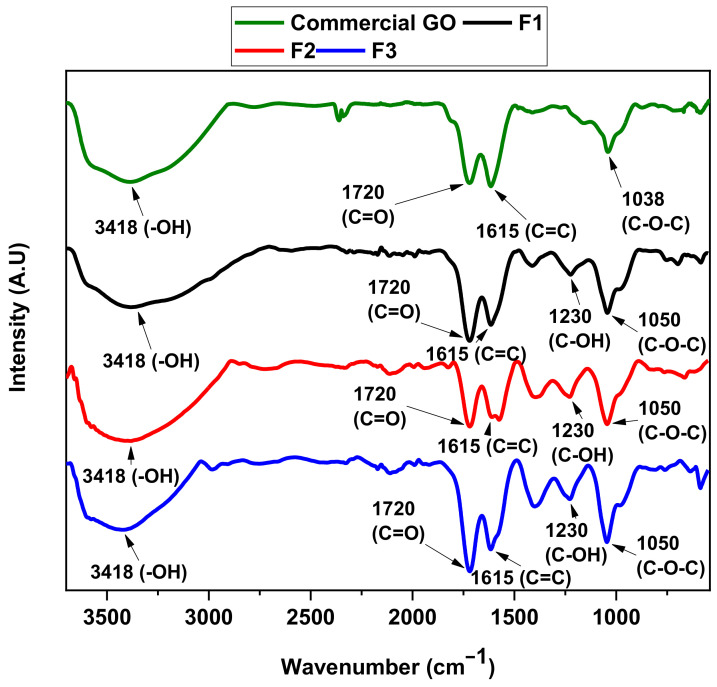
FT-IR of graphene oxide formulations F1, F2, F3, and commercial GO V50 (Standard Graphene Inc., Ulsan, Republic of Korea).

Figure 1 exhibits the bands related to the different GO functional groups, which are also compared with a commercial sample of GO. In all GO samples, a broad band at 3418 cm^−1^ was observed, related to residual water intercalated between the GO films; in addition, it was possible to observe the symmetric vibrations of the C=O group at 1720 cm^−1^ present at the edges of the GO films as well as the presence of the skeletal vibration of the C=C bond of the unoxidized graphene at 1615 cm^−1^. The band at 1230 cm^−1^ is associated with the hydroxyl bending. Finally, the vibrational mode at 1050 cm^−1^ corresponds to the stretching of the epoxy (C-O-C) located on the basal plane of the graphene oxide [19]. 

These samples have comparatively large areas of oxygen-related bands, which indicate a relatively high oxygen content. The FT-IR confirmed the oxygen content as well as other characterization results. Furthermore, the FT-IR spectra results reveal variations in oxidation levels among the formulations. This is evidenced by shifts in the intensity of the C=C band at 1615 cm^−1^ and the C-O-C bond at 1050 cm^−1^. These changes are associated with generating out-of-plane epoxide groups and lactone/ether molecules on the aromatic rings of graphene [20].

### 2.2. Diffractogram for the Different GO

**Figure 2:**
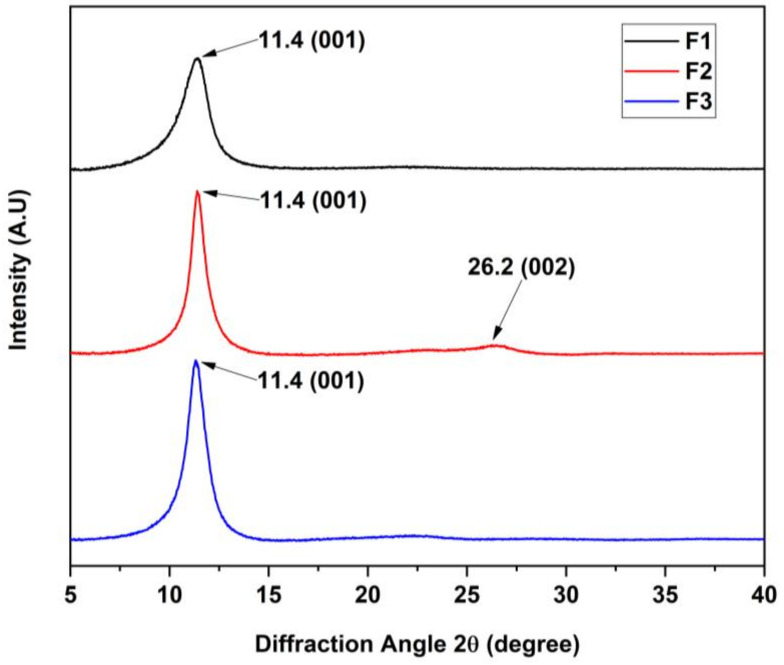
Diffractogram of the different graphene oxides for F1, F2, and F3.

Figure 2 shows the different crystallographic planes for the different GOs. In this diffractogram, it is possible to observe the absence of the 2θ angle at 26.3° attributed to the crystallographic plane (002) of graphite’s hexagonal nature, unlike the F2 formulation, which shows a small contribution of graphite. 

On the other hand, the (001) plane (the hexagonal nature of graphene oxide) produced a peak at a 2θ shift of 11.4°. Moreover, the application of Bragg’s law supported the calculation of the interlaminar distance, where the angle 2θ corresponds to a distance of 7.78 Å. Different authors differ in the interlaminar distance for graphene oxide, which is close to 8 and 10.9 Å. This is probably due to the presence of water molecules bonded to the functional groups on the basal plane of GO, causing an increase or decrease in the distance d [21]. In this sense, it has been concluded that wet GO has a distance of 12 Å, while GO has a distance of 6.1 Å. The XRD findings suggest that the samples exhibit significant oxidation, which is particularly noticeable in the different formulations, where a more substantial presence of adsorbed water accompanies a notable increase in interlaminar distance.

### 2.3. RS for the Different GOs

**Figure 3:**
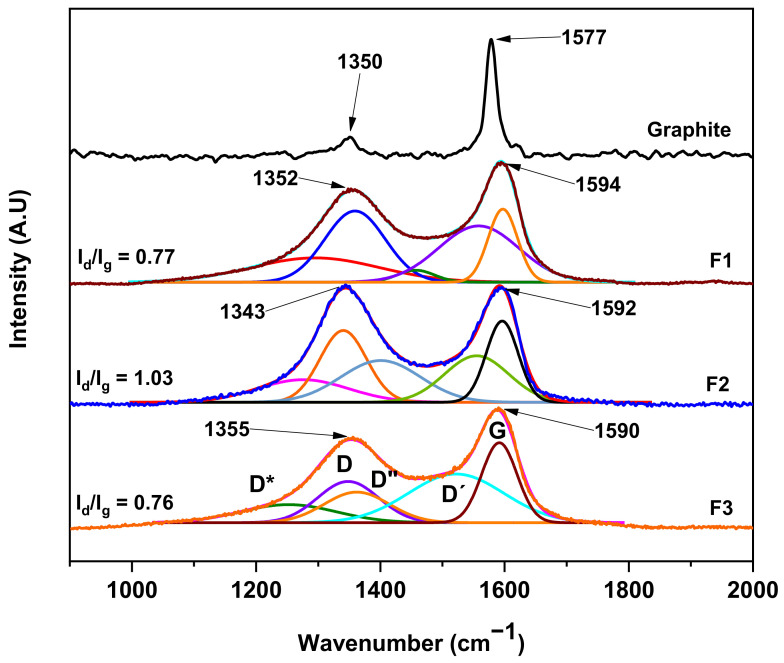
RS for the different graphene oxide F1, F2, and F3 samples.

Raman spectroscopy is essential for obtaining quantitative and qualitative information about graphene oxides because it provides information about the defects formed, layer number, crystallite size, and other aspects. The RS for the GOs was analyzed accordingly (Figure 3). All spectra generally present the two characteristic bands in the first-order region attributed to the D (1350 cm^−1^) and G (1590 cm^−1^) bands. Nevertheless, to allow a more accurate analysis, it is necessary to understand the contribution of the internal band present between the D and G peaks. In this regard, the analysis by RS and its deconvolution allows us to obtain more information through the presence of these bands. 

Some authors have demonstrated that for different GOs, it is necessary to fit the D and G bands through five functions corresponding to the presence of the G, D, D′, the minus-referenced D* (1150–1300 cm^−1^), and D″ (1400–1500 cm^−1^) bands [20,22,23]. Our study observed a strong coherence between linearity (R^2^) and shift square (X^2^) mathematical parameters, as shown in Table 1. Furthermore, we present the sum of the five Gaussian functions, resulting in the overlap between the sum of the Gaussian functions and the RS. 

The D″ band is related to the amorphous phase of the crystal. A reduction in material intensity suggests heightened crystallinity. The D* band relates to the disordered graphitic network resulting from sp^3^ hybridized carbon. Consequently, heightened band intensity indicates the bonding of oxygenated groups on GO sheet edges. Conversely, the D′ band is associated with the material’s surface deformation. Additionally, the D band corresponds to vibrational modes related to functional groups on the graphitic carbon basal plane. The G band, attributed to the E_2g_ mode of first-order dispersion in graphene, is also observable [21,22]. 

On the other hand, the D″ band has been associated with small crystallite size from amorphous phases in GO. Several reports have shown that crystallite size is inversely proportional to the ratio of intensities between the *D* and *G* bands. Therefore, the relative intensities were calculated according to the fitted spectrum and used to estimate crystallite size. In the spectrum, it can be observed that the F2 formulation presents a high *I_D_/I_G_* ratio (1.03) in contrast to the other samples. The *I_D_/I_G_* ratios of the different samples are between 0.77 and 0.76 for F1 and F3, respectively, suggesting that samples F1 and F3 have similar crystallinities but not F2. The crystallite size (La) was determined using the Tuinstra–Koenig model [21].

(1) $$L_{a}=(2.4\times 10^{-10})\lambda^{4}{\left(I_{D}/I_{G}\right)}^{-1}$$

where *λ* is the wavelength of the laser source (nm), and *I_D_* and *I_G_* are the integrated intensities under the *D* and *G* bands, respectively. The estimated crystallite sizes of GO samples F1, F2, and F3 are 25.0, 18.7, and 25.1 nm, respectively. Additionally, alternative ratios have been suggested to determine the degree of organization of the material based on the radius of areas under the curve. In this sense, for the different GOs realized in this work, the bands D*, D, D″ and D′ are the product of the synthesis, and the ratio of modes from defects (non-graphitic modes) to total modes can be determined by the following ratio R′ = (D* + D + D″ + D′)/(D* + D + D″ + D′ + G).

Based on the equation and data in Table 1, formulations F1, F2, and F3 ratios of 0.85, 0.81, and 0.79 are observed, respectively, which suggests that the sample containing a higher oxidation degree is F1, implying that although F3 has a higher amount of oxidizing agent, the oxidation degree is independent of the amount of oxidizing agent as well as the exposure time between the graphite sheets and the reagents. Furthermore, the sp bonds formed due to the oxidation reaction of graphite giving rise to GO are reflected in the appearance of the D band. Nevertheless, since some level of graphitization is present, leading to the presence of the G band, it can be inferred that GO is not found with a monolayer morphology [22].

### 2.4. X-ray Photoelectron Spectroscopy

**Figure 4:**
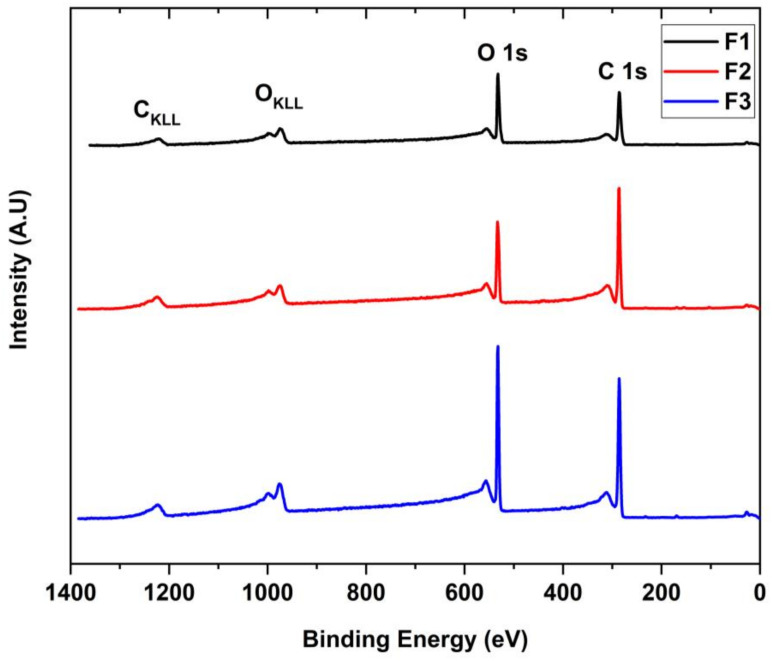
XPS survey spectra of the different GOs for F1, F2, and F3 samples.

X-ray photoelectron spectroscopy (XPS) was used to evaluate the presence of functional groups (Figure 4). In the scanning spectrum, the presence of carbon 1s (286 eV), oxygen O 1s (532 eV), and sulfur (973 eV) is observed. The presence of this last element is probably due to traces left over from the synthesis. 

**Figure 5:**
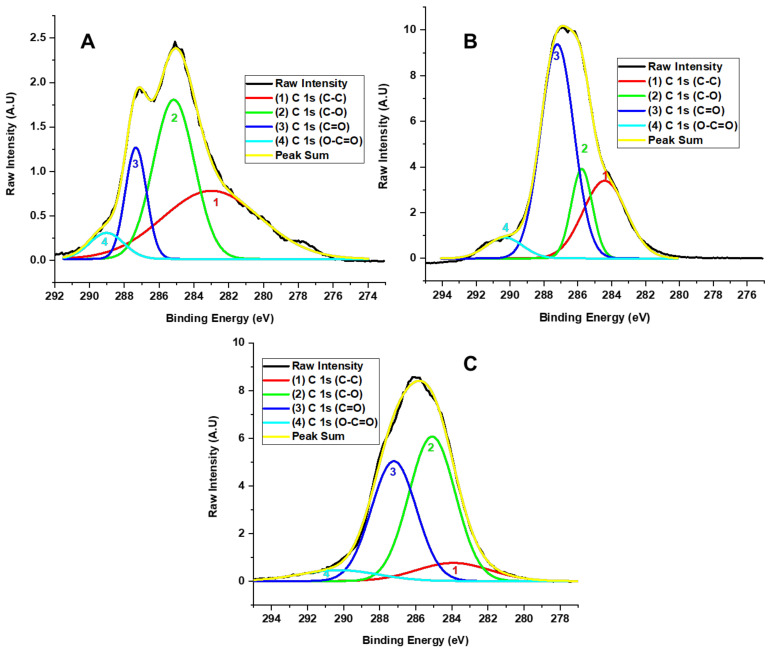
High-resolution XPS spectra for the C 1s of different oxidation state GO F1 (**A**), F2 (**B**), and F3 (**C**) samples.

Figure 5 presents the high-resolution XPS spectra of the C 1s region for the different GO samples. The deconvolution of the C 1s peak revealed contributions from signals with binding energies of 282.99, 285.15, 287.34, 287.33, and 289.01 eV. These correspond to C-C (40.46%), C-O (39.72%), C=O (14.39%), and O-C=O (5.43%), respectively, for the graphene oxide prepared by method F1. Furthermore, the formulation F2 presents one, two, three, and four signals at 284.41, 285.76, 287.211, and 290.32 eV, corresponding to C-C (25.68%), C-O (14.39%), C=O (54.13%), and O-C=O (5.79%), respectively. Thus, the formulation F3 presents the peak deconvolution of the C 1s observed in one, two, three, and four signals, with binding energies at 283.91, 285.09, 287.21, and 290.27 ev related to the bonds C-C (9.35%), C-O (46.93%), C=O (37.66%), and O-C=O (6.06%), respectively. 

**Figure 6:**
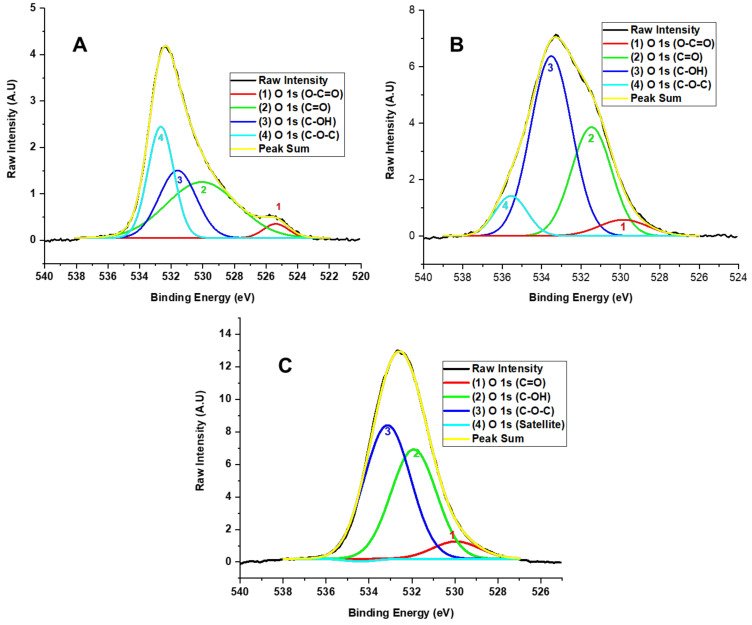
High-resolution XPS spectra for the O 1s region of the different GO F1 (**A**), F2 (**B**), and F3 (**C**) samples.

Figure 6 displays the high-resolution XPS spectrum of the O 1s region. The deconvolution of the O 1s region for method F1 reveals contributions from signals with binding energies at 525.38, 530.06, 531.60, and 532.66 eV, corresponding to the functional groups O-C=O (3.60%), C=O (40.79%), C-OH (25.84%), and C-O-C (29.77%), respectively. Similarly, method F2 shows contributions from signals with binding energies at 529.85, 531.47, 533.51, and 535.56 eV, attributed to O-C=O (5.69%), C=O (31.50%), C-OH (53.50%), and C-O-C (9.31%). For method F3, the spectrum shows signals with binding energies at 529.95, 531.92, and 533.14 eV, related to O-C=O (6.82%), C=O (41.59%), and C-OH (51.59%).

The XPS shows us that the oxidation degree in the samples increases due to the amount of KMnO_4_ and the reaction time. This affirmation is related to decreased C-C bond peak intensity as new oxygenated functional groups, such as hydroxyl, carbonyl, and epoxy groups, increase. In this sense, different oxidation degrees are formed for the various formulations. The high-resolution XPS analysis for carbon in F1 indicated the presence of OH and minor C=O and O-C=O bands. In F2, as the oxidation degree increased, the intensity of the OH band and the O-C=O and C=O bands also increased. In contrast, the band associated with C-C bond energy decreased. In F3, the high-resolution spectrum for oxygen showed a high oxidation degree, with the presence of C-OH, C=O, and C-O-C bonds but without esters. Additionally, the C-O-C band was more prominent than the hydroxyl and carbonyl bands. 

Therefore, we observed hydroxyl and carbonyl groups forming in the XPS related to the F1 and F2 formulation concentrations. Nevertheless, for formulation F3, the formation of epoxide groups prevails, probably attributed to the fact that the Hummers method employs the formation of the dimanganese group (Mn_2_O_7_); as a result, the formation of C-O-C bonds prevails since the dimanganese group supports the epoxidation of the double oxygen bonds from the oxidation of graphite, as observed for F3, which contains a high oxidation degree.

### 2.5. SEM Analysis for the Graphene Oxide Samples

**Figure 7:**
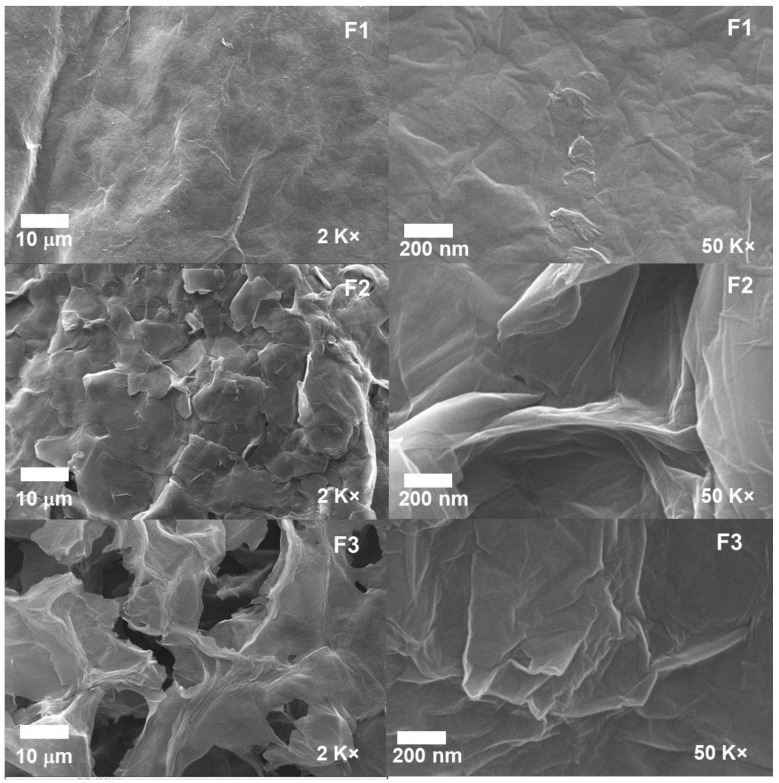
Morphology of the different GO samples F1, F2, and F3.

Figure 7 presents the microstructure analyses for the different formulations. The wrinkled surfaces and folded regions observed in all GO samples are attributed to oxygen-containing functional groups and sp^3^ carbons within the basal planes [24]. Additionally, the different GO samples exhibit varying levels of separation between their graphitic layers, a characteristic attributed to differences in oxidation levels; furthermore, this separation is a consequence of both the oxidation degree and distribution of oxygen atoms between the graphene sheets [23].

On the other hand, the observed morphology is consistent with the data obtained through XRD and RS because the interlaminar distance calculated for the crystallographic plane (001) is 7.78 A, which is related to the absorbed water molecules linked to the functional groups that are found on the plane of the GO. Additionally, in the RS, the presence of the G band can be observed in the formulations F1, F2, and F3, implying that the samples contain a certain degree of graphitization, meaning that the different samples do not exist in the form of a monolayer but rather as a stack of them. Therefore, the presence of separations within the different formulations is consistent with the formulations being between layers but not in a monolayer related to the degree of oxidation of the material.

### 2.6. Histology (In Vivo Studies)

**Figure 8:**
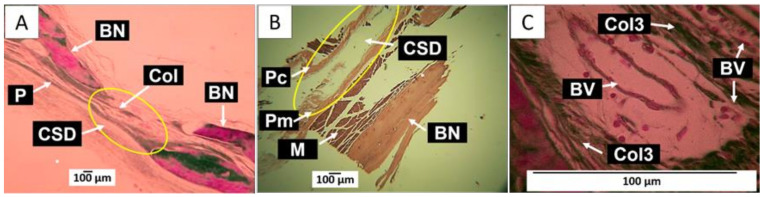
Intracranial implantation in parietal bone of Wistar rat, CSD area (Yellow circle). (**A**) Image at 4× HE technique; (**B**) image at 10× HE technique; (**C**) image at 100× GT technique. P: periosteum; BN: remnant bone; Col: collagen fibers; CSD: critical size defect; Pc: periosteum cranial surface; Pm: periosteum meningeal surface; M: muscle; BV: blood vessel; Col3: type III collagen fibers.

A visual inspection of the intervened areas was performed at the end of the 90 days of implantation and before recovering the samples. In all cases, hair recovery was achieved in the area of the calotte, and the skin was completely healed. Figure 8 corresponds to the images of the control defects, which are left without material. Figure 8A shows that the preparation area healed without bone formation at the expense of fibrous-type scar tissue.

The CSD (critical size defect) was initially defined as the slightest intraosseous preparation that cannot regenerate spontaneously throughout animal life [25]. Still, to date, it is considered that a CSD cannot heal spontaneously through bone regeneration during an experiment [26]. If bone tissue formation occurs, this should not exceed 30% [27]. This work showed that the defects of 5 mm in diameter left as a control did not regenerate spontaneously, behaving as a CSD. It was also observed that the defect healed by forming a fibrous tissue that seemed to come from the periosteum. In contrast, when reviewing the results after implanting GO in the intraosseous preparation, evidence of bone neoformation was found.

**Figure 9:**
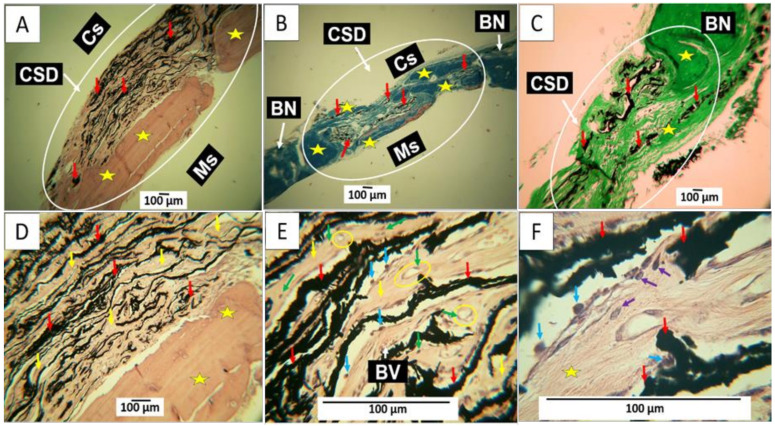
In the intracranial implantation in the parietal bone of Wistar rats, the area of the CSD is implanted with F1. (**A**) Image at 4× HE technique. (**B**) Image at 4× TM technique. (**C**) Image at 4× TG technique. (**D**) image at 10× HE technique. (**E**) Image at 40× HE technique. (**F**) Image at 100× HE technique. Cs: cranial surface of the preparation. White circle: area of interest corresponding to the CSD. Ms: the meningeal surface of the preparation. CSD: critical size defect. Red arrows: remnant GO fragments. Yellow arrow: connective tissue. Yellow star: neoformed bone. BN: remnant bone. Green arrow: osteocytes. Blue arrow: osteoblasts. The yellow circle is an area of histological interest where an osteocyte lacuna appears to form. Purple arrow: osteoblasts that are in the process of converting into osteocytes.

We implanted only two samples of GO (F1 and F2) to consider the effect of the oxidation degree (F1 high and F2 low) on the bone regeneration property, avoiding the excessive use of biomodels. Figure 9 shows the results of the samples implanted with F1. Figure 9A shows that bone neoformation in the lower part of the preparation is in contact with the meningeal membranes (Ms). This area has the appearance of tissue with characteristics of native bone [28,29,30]. The upper part of the defect indicates the presence of GO residues in the connective tissue medium. Figure 9B,C, obtained by Masson and Gomori techniques, respectively, show how the connective tissue is an essential component of collagen fibers type I and III.

The presence of GO immersed in connective tissue is more evident in Figure 9D. The image was taken at a magnification of 10×. Figure 9E corresponds to this same area but at a magnification of 40×, where it is possible to observe that the GO remnants are immersed in a connective tissue that corresponds to bone tissue in formation, with the presence of osteoblasts (blue arrows) and formation of osteocytes and osteocyte lacunae (green arrows), as well as blood vessels (BVs). These cells can be better identified in Figure 9F, in which a magnification of 100× is used.

**Figure 10:**
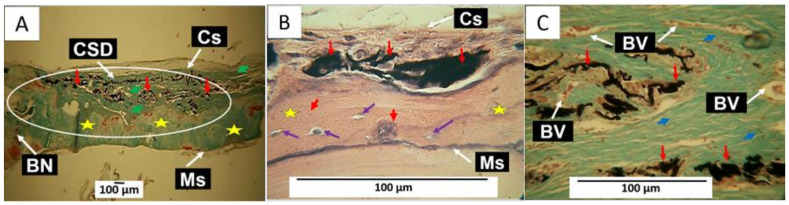
Intracranial implantation in the parietal bone of Wistar rats. The area of the CSD was implanted with F2. (**A**) Image at 4× TG technique. (**B**) Image at 100× HE technique. (**C**) Image at 40× TM technique. Cs: cranial surface of the preparation. Ms: the meningeal surface of the preparation. CSD: critical size defect. Red arrows: remnant GO fragments. BN: remnant bone. Green arrowhead: type III collagen fibers. Red arrowhead: remnants of GO in the process of resorption. Yellow star: neoformed bone. White circle: area of interest corresponding to the CSD. Purple arrow: osteoblast in its cavity. BV: blood vessel.

The results of the F2 implantation show many similarities with those shown for F1. An abundance of neoformed bone tissue was observed in the lower part of the preparation that was in contact with the meningeal surface. This tissue has the presence of osteocyte lacunae with osteocytes and blood vessels. Figure 10A, obtained by the Gomori trichrome technique (GT), shows a significant presence of neoformed bone tissue in the meningeal surface of the preparation. At the same time, the GO (F2) remnants are concentrated in the cranial portion. It can also be visualized that the GO remnant fragments are immersed in a connective tissue composed of type III collagen fibers (green arrowheads), which are green due to the GT technique used.

A more significant amount of neoformed tissue in part of the preparation close to the meningeal periosteum can be explained by the proximity to the meningeal membranes. The meninx is a tissue that covers the brain and is in intimate contact with the cranial periosteum (called meningeal periosteum because of its proximity); it is composed of three layers: the pia mater, the arachnoid, and the dura mater, the dura mater being the outermost and closest to the cranial bones [31]. The dura mater consists of a superficial layer or periosteal dura mater that covers the cranial bones and a lower layer or meningeal dura mater; both are intimately united and have the presence of several venous sinuses [31]. It has been found that this tissue has osteoinduction capacity [32], and its cells can exhibit an osteogenic phenotype [33].

The presence of remnants of un-resorbed material in the upper part of the preparation, in proximity to the superior or cranial periosteum, can be explained by the characteristics of rat cranial bones, which are relatively avascular, possess a very limited microvasculature, originate from the periosteum, and lack the osteone system characteristic of other types of bones [34].

Figure 10B is taken at 100× and corresponds to another biomodel where this material was implanted. It can be observed how the neoformed bone tissue in the meningeal portion of the preparation presents the appearance of a more mature tissue with osteocyte lacunae with osteocytes (purple arrows), without further evidence of GO remnants (red arrowheads). Figure 10C corresponds to a sample processed by the Masson trichrome technique. The coronal portion of the preparation is observed to be occupied by connective tissue with the presence of type I collagen fibers and indicated with arrowheads. These fibers are visualized in blue due to the Masson trichrome stain; some GO remnants and numerous blood vessels are also observed.

In the healing process of the CSD implanted with GO, it is observed that at 90 days, the material is immersed in a connective tissue composed of collagen fibers type I and type III without a fibrous capsule. This finding indicates that the material is biocompatible and shows an affinity for the connective tissue without affecting the bone tissue cells. Although there was no complete regeneration of the CSD at the end of the experiment, it is the formation of a significant amount of neoformed bone tissue, with the appearance of normal tissue characterized by the presence of osteocyte lacunae, which indicates the degree of maturation of this tissue.

In all cases, the neoformation occurred on the meningeal surface, which is explained by the high vascularity of the meningeal membranes, in contrast to the low blood supply that can come from the skin, taking into account that initially, the periosteum was not repositioned on the bone in the area where the intraosseous defects were created. Additionally, the topography of the nanocomposites may have served as a stimulus for their colonization by vessels and cells with osteogenic potential that have been identified in the superficial layer of the meningeal dura mater [33].

As shown in Figure 9 and Figure 10, partial regeneration of the defect was observed in all the preparations implanted with GO. However, F1 showed bone tissue with osteoblasts forming an extracellular matrix and in the process of osteocyte differentiation (Figure 9E,F). The results of the samples obtained with formulation F2 show a more mature bone tissue that already has osteocyte differentiation (Figure 10B) and an abundant presence of blood vessels in the cranial portion (Figure 10C), which help to accelerate the resorption of the GO and the maturation of the connective tissue.

The above is because GO has antibacterial properties related to oxidative stress mechanisms generating reactive oxygen species (ROS) and the photothermal/photodynamic effects of the material [35]. Therefore, GO can fight infections by inhibiting the functions of cellular antioxidant enzymes, causing the generation of an excess of free radicals that damage the lipid membrane and ADN of the cells and consequently maximizing the osteogenic properties because the elimination of bacterial biofilms facilitates the adhesion and proliferation of osteoblasts in tissue-engineered structures [36,37]. Table 2 summarizes information on graphene oxide, the study subject, and its findings.

## 3. Materials and Methods

### 3.1. Materials

All reagents used in this research were purchased from commercial sources. Natural graphite flakes (10 mesh, 99.9%) were purchased from Alfa Aesar (Tewksbury, MA, USA). Sulfuric acid (H_2_SO_4_, 95%), potassium permanganate (KMnO_4_, 99%), and sodium nitrate (NaNO_3_) were purchased from Merck (Burlington, MA, USA). Finally, hydrogen peroxide (H_2_O_2_ 30%) was purchased from Sigma Aldrich (Palo Alto, CA, USA). All reagents used for the synthesis did not require additional purification methods.

### 3.2. Synthesis of Graphene Oxide (GO)

The synthesis of graphene oxide with different oxidation degrees was realized by modifying the Hummers method [23]. Briefly, it was started from 3 g of flake graphite, which was oxidized in the presence of the mixture between KMnO_4_ and NaNO_3_, added slowly over H_2_SO_4_, maintaining a temperature of 10 °C, taking into account that after 24 h of reaction, an excess of KMnO_4_ was added according to the formulation used. Then, the reaction mixture is washed with Milli Q water, slowly adding H_2_O_2_ 3% *v*/*v*, and centrifuged at 5000 rpm. Once washed, the obtained GO is exfoliated in an ultrasonic bath (Branson, Brookfield, CT, USA) for 2 h. Finally, the product is dried in a conventional oven for 24 h at 40 °C (Nabertherm LHT 02/18, Lilienthal, Bremen, Germany). The amounts used for each method are shown in Table 3.

### 3.3. Characterization of GO

#### 3.3.1. Fourier Transform Infrared Spectroscopy

The functional groups present in each GO were recorded through an FT-IR-8400 spectrophotometer (Shimadzu, Kyoto, Japan) with a diamond tip in a wavenumber range between 500 and 4000 cm^−1^ with a resolution of 4cm^−1^, using an attenuated total reflectance accessory.

#### 3.3.2. X-ray Diffraction

Crystallographic planes were determined using a PANalytical X’Pert PRO diffractometer (Malvern Panalytical, Jarman Way, Royston, UK), which utilized copper radiation with wavelengths of Kα1 (1.540598 Å) and Kα2 (1.544426 Å). The diffractometer was operated in the secondary electron mode at 45 kV, scanning over a 2θ range between 5 and 80°.

#### 3.3.3. X-ray Photoelectron Spectroscopy

Various energy values associated with the bonding types formed on the graphene oxide surface were determined using a Specs brand photoelectron X-ray spectrometer (Specs, Berlin, Germany) equipped with a PHOIBOS 150 1D-DLD analyzer (PHOIBOS, Kowloon, Hong Kong, China). This instrument utilized a monochromatic Al source (1486.7 eV, 13 kV, 100 W) with a step energy of 20 eV. Each step comprised a precision of 0.1 eV over 20 cycles.

#### 3.3.4. Raman Spectroscopy

RS values were recorded at room temperature with a ThermoScientific X-ray diffraction smart Raman equipped with a 532 nm laser and a power of 6 mW (Waltham, MA, USA).

#### 3.3.5. Scanning Electron Microscopy 

The surface morphology of graphene oxides was elucidated through a Hitachi TM 3000 scanning electron microscope (Musashino, Tokyo, Japan) in the secondary electron mode using a 20 kV voltage accelerator, with a gold layer to increase the conductivity of the samples.

In vivo biocompatibility study of the different graphene oxide

To study the behavior of the material, the subdermal implantation design and the intraosseous critical defect design were developed in Wistar rats. For the implementation of the subdermal design, nine male Wistar rats, 180 days old and with an average weight of 450 g, were used, and as described variables, the dermal and muscular tissues were examined, as well as the possible affectation to the fatty tissue, muscular tissue, and blood vessels. The inflammatory response to the implanted material after 90 days was recorded using three biomodels per period.

The animals were sedated by applying an intramuscular injection of ketamine/xylazine in doses adjusted to the weight of the biomodels (Ketamine, Blaskov Laboratory, Bogotá, Colombia) and xylazine (ERMA Laboratories, Celta, Colombia). The biomodels were shaved in the upper part of the skull, where an incision was made on the sagittal midline; the soft tissues were separated with a peristome to expose the parietal bones, and two intraosseous preparations of 5 mm diameter and total thickness were made, one on each side of the interparietal suture [45,46]. Three of the preparations were used as empty defect controls (without implant), and in the others, the experimental material was placed; afterward, the soft tissues were closed with resorbable suture (Vicryl 4 zeros, Ethicon, Saude laboratory, Goiânia-Brazil), taking into account that the periosteum did not cover the intervened areas. 

Additionally, once the implantation periods were completed, the samples were recovered using the protocol described for subdermal implantations. The samples were decalcified by immersion in formic acid solution for eight days (TBD-2 Decalcifier, Shandon, Fisher Scientific, Waltham, MA, USA). Once the appropriate degree of decalcification was obtained, histological preparation was performed following the protocol described above. Finally, when the implantation periods were over, the models were sacrificed by intraperitoneal injection of ketamine/xylazine in excess. The tissue samples were recovered and stored in flasks with buffered formalin for 48 h. Then, they were washed with a buffer solution and processed for subsequent histological analysis. The analysis was carried out in triplicate, considering the implementation of the minimum number of rats and the ISO 10993-6 standard [47]. We implanted only two samples of GO (F1 and F2) to consider the effect of the oxidation degree (F1 high, F2 low) on the bone regeneration property. 

The tissue sections underwent histological staining using the hematoxylin–eosin (HE), Masson trichrome (MT), and Gomori trichrome (GT) techniques. Histological images were captured using a Leica DM750 optical microscope and a Leica DFC 295 camera. Subsequently, the images were processed using Leica Application Suite version 4.12.0 software (Leica Microsystem, Mannheim, Germany). All procedures followed the recommendations outlined in the ARRIVE (Animal Research: Reporting of In Vivo Experiments) guide. Notably, there were no deaths of biomodels or post-surgical complications throughout the research. Ethical oversight was provided by the Ethics Committee with Biomedical Experimentation Animals (CEAS) in Cali, Colombia, as per Resolution No. CEAS 006-022.

## 4. Conclusions

A modified Hummers method produced graphene oxides (GOs) with varied characteristics from natural graphite flakes. The oxidation conditions and reactant ratios were also adjusted to yield GO powders with different oxygen contents. The FTIR spectra confirmed the oxidation of graphite, as indicated by oxidation-related bands in all samples, which correlated slightly with the oxygen content determined by XPS analysis. The RS study revealed distinct properties of the GOs, which are reflected in the I_D_/I_G_ ratios. Using the Tuinstra-Koenig model, the crystallite sizes (La) were estimated to range from 18.7 to 25.1 nm.

Deconvolution analysis using RS revealed varying intensities of vibration modes associated with defects arising from the material’s graphitization. The resulting I_D_/I_G_ ratios are 0.85, 0.81, and 0.79 for the three types of GO, which suggests that the sample containing a higher oxidation degree is F1, implying that although F3 has a higher amount of oxidizing agent, the oxidation degree is independent of the amount of oxidizing agent as well as the exposure time between the graphite sheets and the reagents. XPS and XRD analyses further confirmed the presence of various oxygen groups in the GO and the characteristic planes of this type of carbon material. Scanning electron microscopy (SEM) illustrates that the different GO samples exhibit varying levels of separation between their graphitic layers, a characteristic attributed to differences in oxidation levels; furthermore, this separation is a consequence of both the high oxygen content and the intercalation of oxygen atoms between the graphene sheets.

The bone neoformation observed in the cranial defects of critical size indicates that graphene oxide, in addition to being compatible and resorbable, acts as a bridge for bone healing through regeneration, especially for F2, with a lower oxygen content; these results may be attributable to the characteristics of the material that might favor the cell because, in the control defects (voids), bone neoformation was very little and limited to the meningeal area. The observed stimulation of bone neoformation could be explained by the fact that GO has a large surface area and a nanostructured topography that could have positively influenced the adhesion and differentiation of osteogenic cells from the periosteal dura mater.

## Figures and Tables

**Table 1:** A summary of the associations between the areas of the deconvolved bands and their correlation parameters R^2^ and X^2^.

	F1		F2		F3	
Band	A	PB (cm^−1^)	A	PB (cm^−1^)	A	PB (cm^−1^)
D*	50.94	1295	33.7	1275	28.28	1252
D	70.64	1359	55.0	1340	41.19	1348
D″	7.33	1456	54.6	1400	32.71	1362
G	35.11	1597	42.3	1596	46.49	1591
D′	74.26	1558	49.1	1555	76.21	1521
A_D_/A_G_	2.01		0.852		0.886	
R^2^	0.999		0.997		0.998	
X^2^	0.00007		0.00024		0.00014	

A = area; PB = band position; R^2^ = correlation index; X^2^ = deviation.

**Table 2:** GO inclusion in different scaffolds for applications in tissue engineering.

Materials	Study Subject	Findings	Ref
Reduced graphene oxide/hydroxyapatite	Osteogenic differentiation of human mesenchymal stem cells (hMSCs).	The composite between r-GO and hydroxyapatite causes the differentiation of hMSCs and generates excellent bioactivity as a potential osteoinducer.	[38]
GO/N-(3-dimethylaminopropyl)-N-ethylcarbodiimide hydrochloride/N-hydroxysuccinimide.	This study hypothesizes that biocompatible GO–collagen is ideal for constructing osteoinductive and anti-fibrosis effects on tissue engineering chambers for bone tissue engineering.	The in vivo test in rats’ MSCs shows that using these systems increases bone mineralization, promotes osteogenic differentiation, improves angiogenic processes, and confers osteoinductive properties.	[39]
GO/hydroxyapatite/gelatin	This study allows the realization of nanostructured powders to increase the GO reduction rate for forming a 3D structure with excellent mechanical properties.	Well-distributed hap particles facilitate cell growth and proliferation and increase mechanical compressive strength.	[40]
Dexamethasone loaded and reduced graphene oxide-coated multipass caliber-rolled Ti alloy of Ti13Nb13Zr (Dex-rGO-MPCR-TNZ)	Graphene oxide for dental applications.	Dex-rGO-MPCR-TNZ results in the significantly enhanced growth and differentiation of MC3T3-E1 cells into osteoblasts.	[41]
Graphene oxide	GO coating with appropriate characterization triggers osteogenic and odontogenic differentiation in stem cells and porcine bone formation.	The GO coating exhibits good mechanical properties, which is demonstrated by increased resistance to fracture loading.	[42]
Monolayer of graphene		Monolayer graphene presents good biocompatibility properties for craniofacial bone tissue engineering research.	[43]
Composite of graphene oxide with silk fibroin		The composite exhibits excellent properties for applications in regenerative dentistry.	[44]

**Table 3:** The amount used for the synthesis of GO.

Samples	F1	F2	F3	Time (h)
Graphite (g)	3	3	3	-
NaNO_3_ (g)	3	3	3	
KMnO_4_ (g)	3	3	3	24 h of reaction
KMnO_4_ (g)	-	3	3	48 h of reaction
KMnO_4_ (g)	-	-	3	72 h of reaction
H_2_O_2_ 3% (mL)	180	180	180	-
H_2_SO_4_ (mL)	90	90	90	-

## Data Availability

Data will be made available through a request from the corresponding author.
